# The Nutrition Transition in Africa: Can It Be Steered into a More Positive Direction?

**DOI:** 10.3390/nu3040429

**Published:** 2011-04-11

**Authors:** Hester H. Vorster, Annamarie Kruger, Barrie M. Margetts

**Affiliations:** 1 Centre of Excellence for Nutrition (CEN), Potchefstroom Campus, North-West University, Potchefstroom, North-West Province 2531, South Africa; Email: bmm@soton.ac.uk; 2 Africa Unit for Transdisciplinary Health Research (AUTHeR), Potchefstroom Campus, North-West University, Potchefstroom, North-West Province 2531, South Africa; Email: annamarie.kruger@nwu.ac.za

**Keywords:** South Africa, nutrition transition, nutrient intakes, obesity, type 2 diabetes mellitus, cardiovascular disease, THUSA-study, PURE-study, THUSA BANA-study, PLAY-study

## Abstract

The objective of this narrative review is to examine the nutrition transition and its consequences when populations in Africa modernize as a result of socio-economic development, urbanization, and acculturation. The focus is on the changes in dietary patterns and nutrient intakes during the nutrition transition, the determinants and consequences of these changes as well as possible new approaches in public health nutrition policies, interventions and research needed to steer the nutrition transition into a more positive direction in Africa. The review indicates that non-communicable, nutrition-related diseases have emerged in sub-Saharan Africa at a faster rate and at a lower economic level than in industrialized countries, before the battle against under-nutrition has been won. There is a putative epigenetic link between under- and over-nutrition, explaining the double burden of nutrition-related diseases in Africa. It is concluded that it is possible to steer the nutrition transition into a more positive direction, provided that some basic principles in planning public health promotion strategies, policies and interventions are followed. It is suggested that sub-Saharan African countries join forces to study the nutrition transition and implemented interventions on epidemiological, clinical and molecular (genetic) level for better prevention of both under- and over-nutrition.

## 1. Introduction: The Problem and Objectives of This Narrative Review

Many countries in Africa are facing a double burden of nutrition-related diseases, with a co-existence of under- and over-nutrition in the same household, community or population. Hunger and under-nutrition, of especially energy and several micronutrient deficiencies, have not been successfully addressed in Africa, as evidenced by the lack of most African countries to stay on track in reaching the Millennium Development Goals [1]. Simultaneously, the epidemiologic transition described by Omran in the early 1970s [2], is seen in the increased prevalence of obesity and other non-communicable diseases (NCDs) in many African countries [3]. The nutrition transition, defined as the changes in dietary patterns and nutrient intakes when populations adopt modern lifestyles during economic and social development, urbanization and acculturation [4,5] is associated with the documented increases in NCDs. Although changes in dietary patterns over time have been part of man’s history, Popkin and Gordon-Larsen [6] recently emphasized that these changes are presently occurring at a very rapid rate in developing countries and at earlier stages of economic and social development. The result is that the global burden of obesity [6] and other NCDs [7] is shifting towards the poor. The effect of the nutrition transition on disease profiles is further often exacerbated by sedentary and stressful lifestyles, decreased physical activity, increased alcohol consumption and cigarette smoking in urbanizing populations [8].

The purpose of this narrative review is to describe the nutrient intake changes and to examine contributing factors and the consequences of the nutrition transition in Africa, using South African data from rural and urban populations as an example, and to suggest new directions for policy, interventions and research to steer the nutrition transition in Africa into a more positive direction.

## 2. The Nutrition Transition in Africa

Most of the broad adverse changes in dietary patterns during the nutrition transition, described by Drewnowski and Popkin [9], have been confirmed in a detailed comparison of the diets of rural and urban Africans in the THUSA (Transition and Health during Urbanization of South-Africans)-study [10,11,12]. These include decreases in staple foods rich in starch and dietary fiber, increases in foods from animal origin rich in total fat and saturated fatty acids, decreases in plant protein sources such as legumes, and increases in energy-dense snack foods, carbonated sweetened beverages, commercially available alcoholic beverages, as well as added sugar, fats and oils in preparation of food. However, in the North-West Province of South Africa, where the THUSA-study was conducted, increases in fruit consumption, probably because of increased availability and affordability in the urban areas, were observed. MacIntyre [10] showed for example that the changes of maize meal intake (expressed as dry weight per person per day) in adult Africans, from rural to urban areas in 1996-1998, were 136 g to 85 g for men and 122 g to 55 g for women. Red meat increased in men from rural intakes of 48 g per day to 82 g per day in urban areas, while snack foods, carbonated beverages, and fruit (apples, bananas and oranges) appeared in the list of the top 10 foods most consumed by weight per person in urban women.

These dietary pattern changes to more palatable diets containing snack foods, fast and convenience foods, but also more meat and fruit, translated to a nutrient intake pattern in which the macronutrient pattern (total energy, total fat, type of fat, total carbohydrate, dietary fiber, and animal-derived protein) could already be associated with an increased risk of overweight, obesity and other NCDs [11,13] while the improvements in micronutrient intakes in urban subjects (especially of calcium, iron, zinc and some vitamins) did not reach recommended values [14]. It is conceivable that in many overweight and obese subjects, sub-optimal micronutrient intakes could lead to a “double burden” of co-existence of under- and over-nutrition in the same person. It is further conceivable that some of the observed micronutrient deficiencies, such as vitamins with anti-oxidant properties, could contribute to the increased risk of NCDs in these subjects.

**Figure 1:**
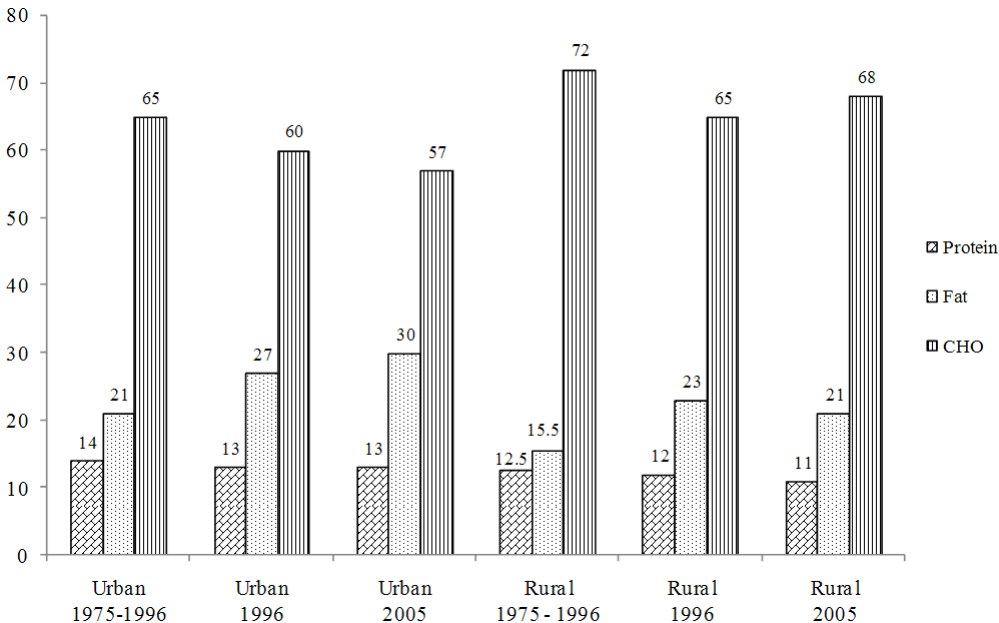
Changes in macronutrient intake as percentage of energy of rural and urban African women in South Africa between 1975 and 2005. The 1975 to 1996 data are from an analysis of the South African literature [15], the 1996 to 1998 data from the THUSA-study [10,11,12,13,14] and the 2005 data from [16] of the PURE (Prospective Urban and Rural Epidemiology)-study.

To illustrate the adverse effects of urbanization on changes in macronutrient intakes of Africans, the contribution of protein, fat and carbohydrate to total energy intake of African women from 1975 to 2005 is given in Figure 1. The figure shows that mean fat intake increased in urban women from approximately 21% of total energy to 30%. The increases in rural women were smaller, from a mean of 15% of total energy to just over 20%. These increases were accompanied by a decrease in mean carbohydrate intake from 65% to 57% of total energy in urban women, and from 72% to 68% in rural women between 1975 and 2005. The increases in protein intake were small, mainly because of an increase in animal protein and decrease in plant protein (data not shown in figure, but summarized in Vorster *et al.* [15]).

**Table 1:** Mean (standard deviation) intakes of selected micronutrients of urban and rural women participating in the THUSA-study (adapted from reference [14]).

Nutrient (unit) Recommended intake (INL98) *	Rural women; Living in deep rural areas	Rural women; From farms	Women living in informal settlements	Urban middle class women	Urban upper class women
	N = 290	N = 148	N = 172	N = 292	N = 105
Calcium (mg)	384 (14)	418 (20)	387 (18)	408 (14)	512 (23)
INL98 = 800					
Zinc (mg)	7.8 (0.2)	7.1 (0.3)	7.6 (0.3)	8.2 (0.2)	10.6 (0.3)
INL98 = 12					
Iron (mg)	8.4 (0.2)	7.5 (0.3)	8.3 (0.2)	10.4 (0.4)	10.4 (0.4)
INL98 = 18					
Vit ** A (RE)	573 (40)	533 (56)	773 (82)	892 (40)	1248 (86)
INL98 = 800					
Vit C (mg)	30 (2)	25 (3)	32 (3)	43 (2)	83 (4)
INL98 = 60					

* INL98: Individual Nutrient Level set at 98%, similar to the American and Canadian RDAs (Recommended Daily Allowances); ** Vit: vitamin; Deep rural areas: former “homelands” under a tribal chief; Farms: commercial farms; Upper class urban: women with professional jobs (teachers, nurses, government officials, *etc.*).

The inability of the dietary changes during the nutrition transition to always meet micronutrient requirement, is illustrated in Table 1, in which mean micronutrient intakes as measured by a validated food frequency questionnaire [10,17] are compared between rural and urban women who participated in the THUSA-study [14]. The table shows that although the “upper class” urban women could meet their vitamin A and C requirements, mean intakes of all groups of rural and urban women of calcium, zinc and iron were well below the recommended levels.

## 3. The Consequences of the Nutrition Transition in Africa

There is no doubt that the nutrition transition, accompanied by decreased physical activity, leads to increases in overweight and obesity [6]. Goedecke *et al.* [18] compared different age groups of adults in 13 sub-Saharan African countries, and showed that South Africa and the Seychelles had groups of women with a mean body mass index (BMI) in the range of overweight (more than 25 kg/m^2^). The differences in overweight and obesity in the different African countries are probably related to economic development. Corinna Hawkes [19] showed in a detailed analysis how globalization and economic development is linked to an obesogenic food environment in middle-income countries. It is clear that production and trade of agricultural goods, foreign direct investment in food processing and retailing and global food advertising and promotion [19] influence diets. Popkin [4] further emphasized that the rate of change in obesity is greater in low and middle-income countries than in higher-income countries.

**Figure 2:**
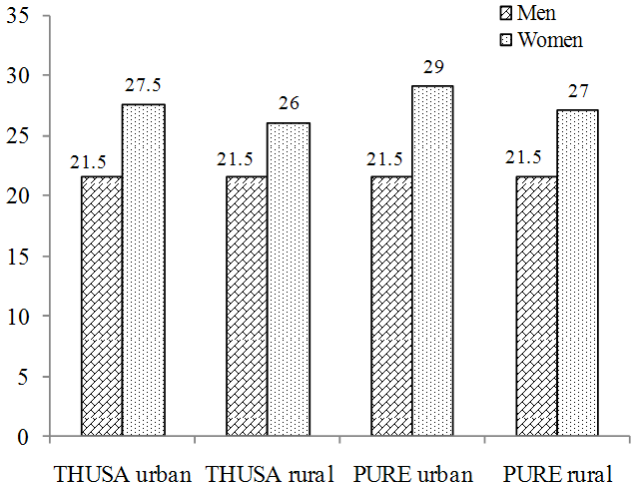
Mean body mass index (BMI) in kg/m2 of urban and rural men and women who participated in the THUSA [11] and PURE [16] studies. A total of 1854 subjects, aged 15 years and older participated in the THUSA-study in 1996-1998 and 2000 subjects, 35 years and older in the PURE-study in 2005.

Puoane *et al.* [20] used the 1998 data from the South African Demographic and Health Survey to show that amongst 5897 African women older than 15 years, 26.7% were overweight with a BMI between 25 and 30 kg/m^2^ while 31.8% were obese with a BMI > 30 kg/m^2^. Therefore, 58.5% of these women were either overweight or obese. The corresponding figure for African men was 25.4%. The same survey showed that 12.9% of African men and 4.8% of women could be classified as underweight, with BMIs < 18.5 kg/m^2^. Clearly, there seems to be a difference in the response to the nutrition transition in adult African men and women regarding weight gain. Figure 2 shows that both the THUSA and PURE (Prospective Urban and Rural Epidemiological)-studies found consistently higher mean BMIs in urban and rural women than in men from the same areas.

The nutritional status of South African children, based on anthropometric indices, is of concern. A national study done in 2005 on 1 to 9 year olds [21] showed that 20% of these children were stunted, 10% were underweight, 10% were overweight and 4% already obese. Goedecke *et al.* [18] mention that the South African Youth Risk Behavior Study, conducted in 2002 and which included more than 9 thousand participants, found that 17% of adolescents were overweight and 4.2% obese. This co-existence of under- and overweight in South African children and high levels of overweight in adult Africans, indicate a complex situation as a result of the nutrition transition that requires specific interventions that will promote optimal nutrition (as will be discussed in section 5).

Overweight and obesity, but also other dietary changes during the nutrition transition, notably increased intakes of total and saturated fat, are associated with an increased risk of NCDs such as stroke, ischemic heart disease, and diabetes [8]. Kruger and coworkers [13] analyzed data from the 1040 apparently healthy women who participated in the THUSA-study and found that age and markers of overweight and obesity such as weight circumference, were significant predictors of serum triglycerides, fasting glucose and HDL-cholesterol while subscapular and triceps skinfolds significantly predicted other NCD risk factors such as blood pressure, serum total and LDL-cholesterol, plasma fibrinogen and the insulin sensitivity index.

Trends in chronic disease mortality has been analyzed for the different South African population groups [22] and clearly demonstrated that adult Africans, who previously had a low risk of NCDs [23], now die from several of these diseases. The 2000 age-standardized death rates showed that of the 1613 deaths per 100,000 in black South Africans, 769 were from chronic diseases, and of these chronic disease deaths in African men and women respectively, 41% and 39% were from stroke, 24% and 16% from ischemic heart disease, 21% and 28% from hypertensive heart disease and 16% and 14% from diabetes.

## 4. The Determinants of the Nutrition Transition in Africa

It is understandable that with economic development, people will choose to follow a more palatable diet than traditional diets high in fiber and low in fat. Mankind has an inherent preference for energy dense, smooth (refined, highly processed), salty, fatty and sweet convenience foods and snacks. Knowing this, the food industry made sure that these kinds of foods are affordable, available, and well advertised and marketed in developing countries [19]. As will be discussed in the next section, this basic knowledge about human behavior and dietary choices must be used in designing preventive measures to steer the nutrition transition into a more positive direction.

**Figure 3:**
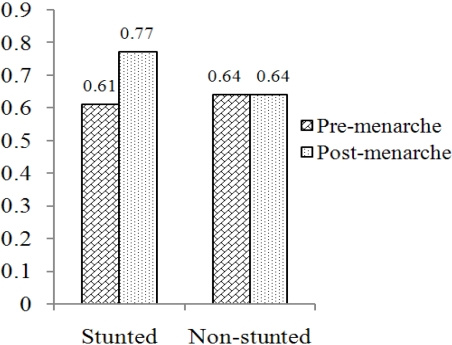
Mean fat mass to body weight ratio of African girls under 12.5 years who participated in the THUSA BANA and PLAY studies [26]. The increase in the fat mass to body weight ratio in stunted girls post menarche was highly significant (p < 0.05).

But it is more difficult to understand why adult Africans, and especially African women, often from poor, food-insecure households, are so vulnerable to obesity when they experience the nutrition transition. Vorster *et al.* [24] has suggested that, based on the Barker hypothesis [25] of fetal programming for vulnerability to NCDs in later life when the expectant mother is nutritionally compromised, stunted children and adults in African households are more vulnerable to obesity when they are suddenly following a modern, “Western” diet. Supporting evidence came from the THUSA BANA (help the children) and PLAY (Physical Activity in the Young)-studies. Figure 3 shows that the ratio of fat mass to body weight in pre-menarche African girls (younger than 15 years) who participated in these studies were similar in stunted and non-stunted girls. However, in the post-menarche period, this ratio increased dramatically and significantly in the stunted, but not in the non-stunted girls [26].

Clearly, there seems to be a “metabolic” contribution to the increased vulnerability to obesity (and other NCDs) when populations who were previously (and still are) struggling with under-nutrition are suddenly exposed to modern energy dense micronutrient-poor diets during the nutrition transition. This is true, not only for South Africa, but also other sub-Saharan African countries. For example, cardiovascular disease is increasing in sub-Saharan African counties [27], while rates of stunting and underweight of 38% and 30% in Nigeria and Kenya are even higher than in South Africa [28].

There are probably also other factors which contribute to this increased vulnerability to the nutrition transition in Africa, such as dietary quality because of the reliance on available and affordable staple foods and energy-dense but nutrient-poor foods, snacks and beverages. For example, the Coca-Cola Company reported in their 2008 annual review [29] that whereas the world average consumption of Coca-Cola in 2008 was 85 servings (a serving being 8 ounces, approximately 240 mL) per capita per year, the consumption in Nigeria was 27 servings, in Kenya 36 servings and in South Africa 252 servings of the beverage.

Another intriguing possibility is that the mechanisms Africans and other developing populations employ to cope with stressful situations, may influence their dietary patterns and response to diet during the nutrition transition. Du Plessis and co-workers [30] recently showed that active and passive coping styles of African men had different influences on their metabolic syndrome indicators. The same group of researchers [31] also showed that Africans with different coping mechanisms had different responses to the handgrip test regarding total peripheral resistance, stroke volume and cardiac output. Furthermore, it is conceivable that the response to the nutrition transition could be influenced by genetic selection and/or epigenetic programming in populations who were previously challenged with survival on low-energy and micronutrient-poor diets, as shown for risk of diabetes in Africans [32,33].

## 5. “New” Directions for Policy and Interventions

The first important approach in designing strategies, policies, and intervention programs to steer the nutrition transition into a direction where under-nutrition and micronutrient deficiencies are addressed, without an increase in the nutrition-related NCDs, is to be positive and to realize that it is (or at least may be) possible. There are examples where this has been accomplished, such as the interventions in North Karelia in Finland, where rural communities of low socio-economic level but high prevalence of NCDs, were targeted with holistic, integrated and well-designed programs [34]. Much can be learned from these and other reported successful interventions, adapting them to local needs and situations. The social drift of the prevalence and burden of NCDs, and particularly of cardiovascular disease also provides circumstantial and indirect evidence that it may be possible to swing the nutrition transition into a more positive direction. With the exception of a few studies on heterogeneous populations, most studies in developing countries indicated that the highest burden of NCDs is found in the higher socioeconomic classes: in employed people with better education and incomes [35,36]. In developed countries, this burden has shifted to poor people with lower socioeconomic status, less education and more unemployment [37,38]. It can be argued that this shift in NCDs, away from the higher socioeconomic classes in developed countries, may be a result of a response to nutrition and lifestyle education, leading to better diets and increased physical activity. It could be argued that the shift may be because those in the higher socioeconomic class can afford better health care. But this is also true for developing countries, where those with the higher NCD burden are also those who probably can afford better health care.

There are, however, some important principles to follow if any of these programs are to be meaningful. A few suggestions are discussed briefly below. They were lifted from a vast literature on nutrition interventions and education, but are by no means the only principles to consider, nor are they in a chronological order to follow when planning interventions. However, following these principles may result in a new direction in which the adverse consequences of the nutrition transition are prevented, while ensuring that all nutrients requirements are met.

1. Plan for holistic, integrated nutrition interventions which will be aligned to all other health promotion campaigns in a country, such as improvement of mother-and child health, anti-smoking, responsible alcohol use, living with a specific condition such as HIV/AIDS or diabetes, school nutrition programs, increased activity initiatives, and others.
2. All interventions should be evidence-based, addressing proven public health problems in the country, with methods known to be potentially successful. A starting point would be to use quality baseline data, scientific theory for planning, and implementation methods to change societal behavior.
3. Under- and over-nutrition should be addressed simultaneously. Interventions should aim for optimal, balanced, adequate, but prudent diets for all. This means that food security for all should be a high priority, and that both the under-nourished and “over-nourished” (overfed) people in a population should be reached with positive messages of how to choose a healthy diet.
4. Involve communities in the planning of the nutrition intervention, using a “bottom-up” rather than a “top-down” approach. Many programs have shown that community ‘buy-in” is necessary for compliance to advice and recommendations.
5. Get all stakeholders on board from the planning stage to ensure that they will help in the implementation of the policy and/or intervention(s). These stakeholders should include local governance bodies, the agricultural and food industries (all involved in production, distribution and marketing of food and beverages), non-governmental organizations, united nations organizations, medical schemes, advertising industry, all institutions providing meals to consumers such as the army, homes for the elderly, schools, *etc*. All these stakeholders are collectively responsible for creating the food environment from which consumers make their choices. They should all work together to ensure an environment in which safe, healthy, cultural and traditional choices are available and affordable. Countries could consider legislation to help to create this environment, such as a ban on advertisements of undesirable foods and beverages on children’s television programs, or a-ban on selling energy-rich, nutrient-poor foods in school canteens. The food industry realizes that there is a market for “healthier foods” but their agenda and motives in developing and marketing these foods are still questionable.
6. Focus on diversification of diets (a food-based approach) rather than a reliance on fortified foods and supplements, where possible. There may be circumstances where fortification and supplementation will be necessary to address specific micronutrient deficiencies, provided that there is good evidence that these deficiencies pose a public health problem. Also, there must be convincing evidence that fortification and supplementation would be safe and effective.
7. It is a human right to be food and nutrition secure. Therefore, in addition of “creating” a suitable food environment, individuals should be enabled to make the right choices. This means appropriate consumer education and social marketing to target groups with strategies that will help to change behavior. Consumer-friendly and understandable food-based dietary guidelines [39] are useful to promote healthy eating and educate consumers on healthy dietary choices.
8. Nutrition “capacity” within the health and other sectors in all African countries should be developed and increased. Even rich, developed, industrialized countries, have insufficient nutritionists and dietitians to “serve” the population. Therefore, nutrition capacity to promote healthier diets can be increased by training other professionals (physicians, nurses, pharmacists, agriculturists, and others) in nutrition.
9. Only implement interventions at scale after thorough field-testing in appropriate target groups. All interventions should be evaluated regularly after implementation to adapt and change if they are not working. These evaluations should include input, process, output, outcome and impact assessments.
10. Use all available resources to implement interventions aimed at healthier eating. Innovative, creative and original use of available infrastructure and existing programs, based on knowledge of local beliefs, attitudes and practices, could ensure positive results.

## 6. Research Challenges for Africa

More information about the dietary patterns and nutrient intakes of different African populations, how these dietary exposures are related to health outcomes, and the forces in the food environment that drives eating behavior in African populations will help to understand and address the nutrition transition in Africa better and more comprehensively. Information is needed on different levels, and the research challenges for Africa are related to this required information. A starting point could be to make sure that quality health statistics in countries are available. The three levels of research needed will be briefly discussed below. Very few Universities and Research Institutions would have the infrastructure and human resources to focus on all levels of research in nutrition. Forming consortiums of research groups could be a solution to increase capacity to tackle nutrition related problems on the suggested three levels.

On the first level, more prospective nutritional epidemiological studies in which dietary intakes are related to risk of NCDs are needed. One such study, the international 12 year Prospective Urban and Rural Epidemiology (PURE)-study, conducted in Africans older than 35 years, has South African, Zimbabwean and Tanzanian cohorts, but more African countries should come on board. These studies could further be integrated with specific interventions in specific communities, which would enable researchers to monitor the impact of certain interventions. Studies on this level could also focus on the determinants of eating behavior in different cultures. The complexity of factors influencing behavior dictates that these studies should be trans-disciplinary, in which researchers from a variety of scientific disciplines work together to solve problems. We need to know more about these factors and determinants of eating behaviour in Africans experiencing the health and nutrition transition. Psychological knowledge and skills are necessary to investigate them in-depth.

On the second, clinical level, more research on how Africans respond to different dietary interventions aimed at primary and secondary prevention of NCDs and their risk factors are needed. It is not known if dietary recommendations to prevent risk factors for NCDs are the same in different populations. More randomized controlled trials are needed with tightly controlled dietary intakes to define optimal, culturally-friendly diets for prevention and treatment of NCDs in specific groups of Africans.

On the third, molecular level, mechanistic or “experimental” studies could help to understand the genetic, epigenetic and molecular mechanisms which are responsible for differences in individual and/or groups responses to the nutrition transition. It could also shed more light on the double burden of co-existence of under- and over-nutrition. It is possible to combine these studies with those on the first two levels, collecting the genetic and other biological samples in those studies for further laboratory analyses. With limited resources for research in sub-Saharan Africa, it is suggested to build the infrastructure for these type of studies only in a selected few centers. These Centers could then form a platform or hub of a network of African researchers working together to steer the nutrition transition into a positive direction.

## 7. Conclusions

This narrative review has shown that the nutrition transition in sub-Saharan African countries is complex, because over-nutrition associated with overweight, obesity and other NCDs emerged before the problems of under-nutrition and micronutrient deficiencies have been solved. However, it is concluded that the nutrition transition may be steered into a more positive direction if the suggested basic principles are used in the planning of holistic, integrated policies and interventions aimed at optimal nutrition for all. It is suggested that sub-Saharan countries join research efforts, as well as human and infrastructure resources, for a better understanding of the nutrition transition, in order to design interventions that will be tailor-made for Africa. Furthermore, it is crucial that sufficient human capacity is developed in Africa to implement these policies in appropriate intervention programs.
